# Effect of potassium nitrate on tooth sensitivity during in-office bleaching: A randomized clinical trial

**DOI:** 10.4317/jced.61765

**Published:** 2025-02-01

**Authors:** Deise Caren Somacal, Melissa Castro do Rio, Hélio Radke Bittencourt, Luiz Henrique Burnett Júnior, Ana Maria Spohr

**Affiliations:** 1 Department of Restorative Dentistry, Pontifical Catholic University of Rio Grande do Sul, Porto Alegre, Rio Grande do Sul, Brazil; 2 Department of Statistics, Pontifical Catholic University of Rio Grande do Sul, Porto Alegre, Rio Grande do Sul, Brazil

## Abstract

**Background:**

The study is a triple-blind, split-mouth, randomized trial to evaluate and to compare the effect of different concentrations of potassium nitrate (PN) prior to in-office bleaching on tooth sensitivity (TS).

**Material and Methods:**

Sixty participants were randomly divided into groups (n=30): Group A: 5% PN (positive control) and 10% PN; and Group B: 5% PN (positive control) and 35% PN. Each concentration of PN was applied to a hemi-arch for 10 min, and then a 35% bleaching agent was applied. The participants underwent two sessions of bleaching with a one-week interval. TS was recorded using a questionnaire and a visual analog scale.

**Results:**

According to McNemar’s test, there was a significant reduction in the prevalence of TS in group A (*p*=0.013) and group B (*p*=0.000) across the time assessments. Fisher’s Exact Test showed no significant difference between the control and treatment sides (*p*>0.05). There was a significant reduction in TS intensity for both groups (*p*<0.05).

**Conclusions:**

The 10% and 35% PN were effective in reducing the prevalence and intensity of TS, as well as 5% PN, and the treatments did not affect color change.

** Key words:**Clinical trial, dentin sensitivity, tooth bleaching, hydrogen peroxide, potassium nitrate.

## Introduction

The mechanism of action of the bleaching agent is directly related to hydrogen peroxide that breaks down long molecular chains of pigments through a reaction of oxide reduction, releasing free radicals (1). These free radicals diffuse through the dental structure due to their low molecular weight, being able to reach inside the pulp tissue within a few minutes after application (2).

The interaction of the bleaching agent free radicals with the tooth-pulp complex may modify the pulp’s condition. Its adverse effect is a temporary mild pain for the patient and a high prevalence of tooth sensitivity (greater than 87%) (3,4). Furthermore, previous studies show its influence on the patients’ quality of life (5), as it ranges from being mildly uncomforTable to becoming a reason for giving up the treatment altogether (3,5).

Among the frequently observed pulp alterations, the neurogenic inflammation through activation of the TRPA1 ion channel is noteworthy (6), which releases the *P* substance that acts on pain modulation, increasing vascular permeability and vasodilation (7). However, the mechanism that generates dental sensitivity by peroxide action remains unclear. Hydrodynamic theory which justifies dentin hypersensitivity is still the most accepted theory as the explanation for this adverse effect (3).

Analgesics and anti-inflammatory drugs were used to control sensitivity caused by in-office bleaching (8,9), but the use of these drugs did not reduce the prevalence and intensity of sensitivity (10). However, there are methods that could reduce sensitivity after tooth bleaching based on the use of topical desensitization agents or addition of these components into the bleaching gels and toothpastes (11). Their mechanism of action may be classified under two types of desensitization agents: neural agents (direct action on nerve endings) and obliterating agents of dentinal tubules. The only known desensitizing agent of neural action is potassium, which acts by either reducing receptor excitability or interrupting neural transmission through potassium ions depolarization (12,13). In addition, potassium is often associated with oxalates and nitrates (13,14).

The use of 5% potassium nitrate as a topical desensitizer reduced tooth sensitivity after bleaching with 35% hydrogen peroxide (15,16). Nevertheless, the use of 10% potassium nitrate gel resulted in a 35% reduction in dentin hypersensitivity within 48 to 96 hours (17). The use of the same neural agent at higher concentrations (35%) resulted in a 91% immediate decrease in dentin hypersensitivity (18). Then, it is reasonable to consider that higher concentrations of potassium nitrate could reduce sensitivity immediately after tooth bleaching, as in dentin hypersensitivity case. To the best of the author’s knowledge, there are currently no studies comparing high percentages of potassium nitrate (10% and 35%) as a desensitizer agent during in-office bleaching.

Therefore, the aim of this clinical trial was to evaluate and to compare the effect of potassium nitrate 5% and potassium nitrate in different concentrations (10% or 35%) with 2% sodium fluoride prior to in-office bleaching with 35% hydrogen peroxide gel on the tooth sensitivity. The null hypothesis was that potassium nitrate in different concentrations will not influence the tooth sensitivity.

## Material and Methods

-Ethics approval

This clinical trial was approved by the local ethics committee (06581219.2.0000.5336).

-Protocol registration 

This study was registered in the Brazilian Registry of Clinical Trials (REBEC) under the identification number RBR-3p54y6r. This report follows the protocol established by the CONSORT statement (19).

Trial design, and locations of data collection

This was a randomized, split-mouth, triple-blind (participants, operator, and examiners) trial with an equal allocation rate between groups. Participants were recruited and treated from August 2019 to December 2020. Due to the Covid-19 pandemic, all participants were assisted in a private office. The participants signed an informed consent form.

-Recruitment and eligibility criteria

Inclusion criteria of participants: at least 18 years old; good general and oral health; a minimum of 24 teeth and no restorations in incisors, canines and premolars; and color greater than A2, B2, C2 or D2 as assessed by a value-oriented shade guide (Vita Classical, Vita Lumin, Vita Zahnfabrik, Bad Säckingen, Germany). Factors taken into consideration for the exclusion of potential participants included: previously tooth-bleaching procedures; pregnancy or lactating; teeth with cracks, cervical lesions; exposed dentin or a history of dentin hypersensitivity; previous (last three months) use of desensitization agent or anti-inflammatory drugs; and any oral pathology. The experience of tooth sensitivity was evaluated in the participants through a jet of air 1 mm from the tooth, in the cervical third of the clinical crown.

-Sample size calculation

Sample size was based on the absolute risk of the primary outcome of the study (tooth sensitivity). A significance level of 5% and a nominal power between 80 and 90% was stipulated. Thus, 60 participants were required, 30 participants for group A and 30 participants for group B.

-Random sequence generation and allocation concealment

**Figure 1:**
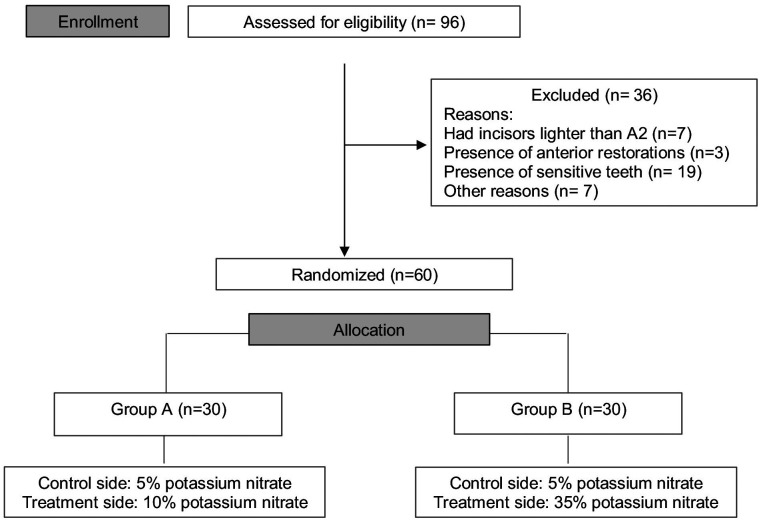
Flow diagram of the clinical trial including detailed information on the excluded participants.

The participants were selected and randomly divided into two groups, A and B. The application of the desensitizer gel was through “split mouth” design in order) to test three different concentrations: 5% potassium nitrate (positive control side) in both groups in one side of the mouth, and 10% potassium nitrate (group A) or 35% potassium nitrate (group B) in the other side of the mouth (Fig. 1). A third operator, not involved in the research protocol, was responsible for the randomization procedure by using 30 opaque and sealed envelopes which contained the description of the technique to be performed on the participant. Each envelope involved two participants, as the second participant received the opposite treatment from the first participant. Participants and operators were blinded from the groups to which the participants were allocated.

-Study intervention

The materials used in the study are in  Table 1.

All participants received prophylaxis with a mixture of pumice and water, applied with a Robinson brush. After placement of a lip retractor, a polyester strip was placed between the upper and lower central incisors. A 1.0 ml syringe with a plastic tip was used to apply the 5%, 10% or 35% potassium nitrate gel on both arches. On each arch, an amount of 0.5 ml of gel was applied, forming a layer of approximately 2 mm thickness per tooth. Both syringes (positive control and treatment gels) were labeled with the patient’s name and application side so that operator, patient and evaluator were blinded for the procedures applied:

Group A - 5% potassium nitrate + 2% sodium fluoride was applied on one side of the mouth, while 10% potassium nitrate + 2% sodium fluoride was applied on the other side of the mouth for 10 min without rubbing or stirring.

Group B - 5% potassium nitrate + 2% sodium fluoride was applied on one side of the mouth, while 35% potassium nitrate + 2% sodium fluoride was applied on the other side of the mouth for 10 min without rubbing or stirring.

After 10 min, the desensitizer gel was removed with an ejector aspirator, rinsed, and dried with air jet. A light-cured gingival barrier (Top Dam, FGM, Joinville, SC, Brazil) was applied from the second right premolar to the second left premolar in both arches. After that, the 35% hydrogen peroxide (Whiteness HP Blue, FGM, Joinville, SC, Brazil) was mixed with the thickener just before its application. For each participant, a syringe of hydrogen peroxide and a syringe of thickener was used in each session, uniformly distributing the entire contents of the mixture in both arches. A single application of the 35% bleaching gel for 40 min was made per session, following the manufacturer’s recommendations. Afterwards, the bleaching gel was removed with an ejector aspirator and abundantly rinsed. The participants underwent two sessions with an interval of one week, being instructed to brush their teeth using non-desensitizing fluoride toothpaste (Colgate Maximum Neutral Protection, Colgate Palmolive Company, SP, Brazil) and soft toothbrush. These products were provided by the research investigator for standardization.

Tooth sensitivity evaluations

Each participant received three copies of the same questionnaire which contained the following questions: 1) Did you feel tooth sensitivity?; 2) If you answered “yes” to the previous question, was there a difference in sensitivity between the sides of the mouth?; 3) If you answered “yes” to question 2, on which side did you feel more sensitivity? The first copy of the questionnaire was answered at the end of the day of the first bleaching session (day 1); the second copy was answered at the end of the day of the second bleaching session (day 7); the third copy was answered seven days after the second bleaching session (day 14). The participants were instructed to complete the questionnaires at similar times (6 pm) on the dates which were previously defined (day 1, 7 and 14). Participants also reported their perception of tooth sensitivity using a visual analogue scale (VAS) (0 = no pain to 10 = intolerable pain). The results were organized into two categories: a) absolute risk of tooth sensitivity, and b) intensity of tooth sensitivity.

-Statistical analysis 

The comparison of the absolute risk of tooth sensitivity across time assessments for each group (A and B) was performed using the McNemar’s test. The Fisher Exact Test was used to compare the side of the mouth with greater sensitivity (positive control and treatment) between groups (A and B). The comparison of tooth sensitivity intensity across time assessments in each group (A and B) was performed by applying Wilcoxon test. Mann-Whitney test was used to compare the tooth sensitivity intensity between groups A and B in each time assessment. The significance level was preset at 005.

## Results

-Characteristics of included participants

A total of 96 participants were preliminarily examined to identify those which met the requirements to participate in this study. Of these, a total of 60 participants were selected and included in this clinical study (Fig. 1). Forty-eight percent of the participants were men. The average age of the participants was 25 years old.

-Adherence to the protocol and drop-outs

No participant discontinued intervention.

-Tooth Sensitivity

 Table 2 presents the absolute risk of tooth sensitivity. The reduction in the prevalence of tooth sensitivity between day 1 and day 7 was significant according to McNemar’s test in group A (*P* = .013) and group B (*P* < .001). In both groups there was a significant reduction in the prevalence of tooth sensitivity between day 1 and day 14 (*P* < .001). No participant reported tooth sensitivity at day 14.

Fisher’s Exact Test showed no significant difference between the control and the treatment sides between groups at day 1, day 7 and day 14 (*P* > .05) ( Table 3).

There was a reduction in tooth sensitivity intensity across the time assessments for groups A and B. Wilcoxon test showed no significant difference in tooth sensitivity intensity between day 1 and day 7 in group A (*P* > .05). However, significant differences were observed in tooth sensitivity intensity between day 7 and day 14 (*P* < .001), and between day 1 and day 14 (*P* < .001). In group B, there was a significant difference in tooth sensitivity intensity for the comparison of all time assessments (*P* < .05) ( Table 4).

According to Mann-Whitney test, there was no significant difference in tooth sensitivity intensity between groups A and B in each time assessment (*P* > .05) ( Table 5).

-Adverse effects

No participant reported an adverse effect other than tooth sensitivity.

## Discussion

The absolute risk of tooth sensitivity at the first session was high in both groups. These results are in agreement with other studies (9,20) and can be justified by the high concentration of hydrogen peroxide used in the bleaching procedure (21). Hydrogen peroxide reaches the pulp tissue within a few minutes after application (22). In the pulp tissue, it causes an inflammatory reaction, oxidative stress and cell damage (7). These processes release adenosine triphosphate, prostaglandins and other inflammatory mediators that stimulate nociceptors (16,23,24). The use of potassium nitrate as a desensitizer has been described as a good alternative to minimize this side effect, because it not only reduces the dentinal sensory nerve activity, but also prevents nerve fiber repolarization due to the presence of positively charged potassium outside the nerve membrane (24-26).

Although the absolute risk of tooth sensitivity decreased over the days in the present study, Fisher’s exact test showed no significant difference between the positive control side (5% potassium nitrate) and the treatment side (10% or 35%) in both groups. This result supports the hypothesis of the present study by showing that different concentrations of potassium nitrate did not influence tooth sensitivity. However, the pain analysis is subjective, and the pain threshold of each participant may have influenced the responses to the questionnaire. The participant´s perception of pain in relation to the side of the mouth with greater sensitivity is difficult to measure, given the participant´s lack of calibration to fill out the questionnaires, as well as their inexperience regarding the sensation of tooth sensitivity, since they have never received tooth whitening and had no history of dentin hypersensitivity.

Potassium nitrate at 5% was applied as a positive control group on one side of the split mouth, in both groups. This concentration and its use in commercial form (not manipulated) was chosen based on promising results previously demonstrated in the literature (12,15). It is important to emphasize that a placebo group was not used in the present study. This decision was based on ethical issues, since it is known that tooth sensitivity occurs during the dental bleaching treatment and that there are procedures to reduce this discomfort (15). On the treatment side, 10% or 35% potassium nitrate was used for groups A and B, respectively. These concentrations were also chosen based on results found in the literature for dentin hypersensitivity (17,18). Therefore, the present study followed the split-mouth design, based on other studies with similar subjects (27). That has the advantage of removing a lot of inter-individual variability from the estimates of the treatment effect as the patients act as their own controls. Possible disadvantages of a split-mouth designs are related to carry-cross effects, contamination or spilling of the effects of one intervention to another (28). To avoid these disadvantages, a polyester strip was placed between the upper and lower central incisor to avoid contact of the desensitizer gels. In addition, the gels were carefully and completely removed from the teeth surface using an ejector aspirator, followed by rinsing and drying.

Associated with potassium nitrate, 2% sodium fluoride was added to the desensitizers manipulated in the present study in order to match the composition of the control desensitizer, which contains 2% sodium fluoride. Sodium fluoride is one of the most commonly used agents in the treatment of dentin hypersensitivity (14). Association of potassium nitrate and sodium fluoride to reduce tooth sensitivity caused by tooth bleaching still does not have its mechanism of action well understood. Potassium ions as active components of potassium nitrate work to reduce pulp sensory activity through depolarization of K+ (18,24,25), functioning as a neural agent. Taking that into account, it is imperative that the neural agent manages to reach the pulp chamber before the penetration of hydrogen peroxide. It was observed the penetration of potassium nitrate into the pulp chamber of a bleached tooth 30 min (2,29) and 5 min (2) after application. These findings explain the methodological choice of the present study in carrying out the application of the desensitizer prior to the use of hydrogen peroxide.

Some authors reported that the application of high concentrations of potassium nitrate on the tooth surface can generate a high osmotic gradient. This would result in the movement of dentinal fluid and consequent stimulation of mechanoreceptors located in the dental pulp. Thus, pain sensitivity could occur not only from the inflammatory process generated by hydrogen peroxide, but also from the movement of dentinal fluid (30). The high concentrations of potassium nitrate used in the present study may not have negatively affected sensitivity due to the presence of another active desensitizing agent, 2% sodium fluoride.

Despite the limitations of the study, it was concluded that the 10% and 35% concentrations of potassium nitrate associated with 2% sodium fluoride were effective in reducing the prevalence and intensity of tooth sensitivity over the two-week evaluation period, as well as 5% potassium nitrate.

## Figures and Tables

**Table 1:** Materials used in the study.

Material	Composition	Manufacturer
Whiteness HP Blue 35 Whitening Agent	35% hydrogen peroxide, thickeners, inert violet, neutralizing agent, calcium gluconate, glycol and deionized water	FGM Dental Products, Joinville, SC, Brazil
Top Dam Light-cured resin dam	HEMA, urethane di-methacrylate monomers, inert filler, pigments and photoinitiators.	FGM Dental Products, Joinville, SC, Brazil
Desensibilize KF 2% Desensitizing control agent	Active agents – 5% potassium nitrate, 2% sodium fluoride Inactive agents – deionized water, glycerin, neutralizing agent and thickeners	FGM Dental Products, Joinville, SC, Brazil
Manipulated desensitizing agent	Active agents – 10% potassium nitrate, 2% sodium fluoride Inactive agents – deionized water, glycerin, neutralizing agent and thickeners	School of Health and Life Sciences PUCRS, RS, Brazil
Manipulated desensitizing agent	Active agents – 35% potassium nitrate, 2% sodium fluoride Inactive agents – deionized water, glycerin, neutralizing agent and thickeners	School of Health and Life Sciences PUCRS, RS, Brazil

**Table 2:** Comparison of the number of participants who experienced tooth sensitivity (TS) across time assessments in each group (A and B).

Time	Day 1		Day 7		Day 14		P value	
	Number of participants with TS							
	Yes	No	Yes	no	Yes	no		
Group A	15	15	5	25	0	30	Day 1 – day 7	0.013
							Day 1 – day 14	< .001
Group B	22	8	0	30	0	30	Day 1 – day 7	< .001
							Day 1– day 14	< .001

Absolute risk of TS using McNemar’s test (α=0.05)

**Table 3:** Comparison of the number of participants with tooth sensitivity between groups (A and B) with regards to the side of the mouth with greater sensitivity (control and treatment) between the time assessments.

	Group A		Group B		P value
	5% PN	10% PN	5% PN	35% PN	
Day 1	13	6	8	11	0.191
Day 7	6	3	1	3	0.266
Day 14	0	0	0	0	1.000

Fisher’s exact test (α =0.05) PN: Potassium nitrate

**Table 4:** Comparison of the medians of scores of tooth sensitivity intensity (0-10) experienced by participants across the times assessments within each group.

		Group A		Group B	
		Median	P value	Median	P value
Control side	day 1 - day 7	2	0.130	2	0.001
	day 7 - day 14	0	< .001	0	0.001
	day 1 - day 14	0	< .001	0	< .001
Treatment side	day 1 – day 7	1	0.480	2	< .001
	day 7 - day 14	0	< .001	0	0.004
	day 1 - day 14	0	< .001	0	< .001

Wilcoxon-paired test (α=0.05)

**Table 5:** Comparison of the medians of scores of tooth sensitivity intensity (0-10) experienced by participants between the groups in each time assessment.

	Time	Group A	Group B	P value
		Median	Median	
Control side	Day 1	2	2	0.903
	Day 7	0	0	0.079
	Day 14	0	0	1.000
Treatment side	Day 1	1	2	0.266
	Day 7	0	0	0.179
	Day 14	0	0	1.000

At each period, the two groups were compared with Mann-Whitney test (α=0.05)

## Data Availability

The datasets used and/or analyzed during the current study are available from the corresponding author.
